# Patient‐Reported Outcomes Following Systemic Antibiotic Adjunct to Nonsurgical Treatment of Periodontitis: A Randomized Controlled Clinical Trial

**DOI:** 10.1002/cre2.70067

**Published:** 2025-01-13

**Authors:** Parastoo Parhizkar, Jaber Yaghini, Omid Fakheran

**Affiliations:** ^1^ Department of Periodontics, Dental Implants Research Center, Dental Research Institute, School of Dentistry Isfahan University of Medical Sciences Isfahan Iran; ^2^ Division of Oral Surgery and Orthodontics, Department of Dental Medicine and Oral Health Medical University of Graz Graz Austria

**Keywords:** antibiotic, health‐related quality of life (HRQOL), periodontitis

## Abstract

**Objectives:**

Considering the importance of patient‐centered care, we aimed to evaluate the impact of systemic antibiotics on oral health–related quality of life during nonsurgical periodontal treatment. This controlled trial addresses a gap in understanding how systemic antibiotics influence patient‐reported outcomes, focusing on Stage III periodontitis.

**Materials and Methods:**

Sixty‐one adults participated in a double‐blind, randomized clinical trial, with participants divided into two groups: the test group, which received antibiotics, and the control group. All the participants received nonsurgical periodontal treatment. We conducted follow‐up assessments at one and 3 months posttreatment, including recording clinical parameters and administering the Oral Health Impact Profile‐14 (OHIP‐14) questionnaire.

**Results:**

The results showed a notable improvement in the quality of life for patients in the test group compared to the control group at 1 month (*p* value = 0.012) and 3 months (*p* value = 0.014) after treatment. While there were improvements in pocket probing depth, gingival index, and clinical attachment loss in both groups, it is worth noting that only bleeding on probing exhibited a significant improvement in the test group after 3 months compared to the control group (*p* value = 0.008).

**Conclusions:**

In summary, incorporating systemic antibiotics alongside nonsurgical periodontal treatments appears to bring about positive outcomes for individuals dealing with Stage III periodontitis during nonsurgical treatment, ultimately enhancing their oral health‐related quality of life.

**Trial Registration:**

Iranian Registry of Clinical Trials (IRCT Id): IRCT20201221049786N1.

## 1 Background

Periodontal diseases, which include gingivitis and periodontitis, are highly prevalent in adults and are mainly caused by intraoral biofilms containing periodontal pathogenic bacteria such as Porphyromonas gingivalis and Prevotella intermedia (Trindade et al. 2023). Although periodontitis is considered a “silent disease,” it causes swelling, bleeding, pain, and tooth loss (Buset et al. 2016).

Since periodontitis is often infectious and dependent on pathogenic microorganisms and most patients cannot mechanically remove microbial plaque, clinical interventions are recommended for more extended periodontal survival (De la Rosa et al. 1979; MacGregor, Rugg‐Gunnand, and Gordon 1986; Kocher, Tersic‐Orth, and Plagmann 1998). Supra‐ and subgingival instrumentation, various types of surgical interventions, patient motivation, and re‐instruction during the maintenance phase of treatment can all be part of periodontitis treatment (Herrera et al. 2022).

Based on the microbial etiology of periodontitis, systemic administration of antibiotics is considered an adjunct to controlling bacterial infections (Barça, Çifçibaşı, and Çintan 2015). According to reports, using a combination of Metronidazole (250 mg) and Amoxicillin (500 mg) three times a day for 7 days has been considered a sensible choice. However, it is deemed ineffective without mechanical treatments (Heitz‐Mayfield 2009).

In recent decades, healthcare systems have increasingly acknowledged the critical role of patients' perspectives in ensuring the delivery of high‐quality, equitable, and safe services. Central to this paradigm shift has been the growing integration of patient‐reported outcomes (PROs), which provide direct insights into patients' perceptions and are now recognized as essential metrics for improving care.

PROs have gained significant attention in clinical dentistry, reflecting a shift towards patient‐centered care. This trend emphasizes the importance of understanding how oral health impacts overall well‐being, beyond just clinical measures like DMFT or periodontal pocket depth. Researchers are increasingly focusing on the psychosocial effects of oral conditions, such as pain, appearance, and social interactions. As a result, PROs have become key metrics for evaluating treatment outcomes and improving patient care in both clinical and public health settings (Williams et al. 1998; Sischo and Broder 2011; Yu et al. 2023).

A key area where patient‐reported outcomes have become particularly valuable is in assessing Oral Health–Related Quality of Life (OHRQoL). OHRQoL is a multidimensional construct that reflects the impact of oral health on an individual's overall well‐being, including physical, emotional, and social dimensions. It goes beyond clinical indicators to capture the subjective experience of patients, such as pain, discomfort, and functional limitations related to oral health (Rothman et al. 2007). Assessing the OHRQoL can help develop policies and interventions to improve patients' health (Nagarajan and Chandra 2012).

Accordingly, previous studies have demonstrated that periodontal treatments can significantly enhance the quality of life in patients suffering from periodontitis (Needleman et al. 2004; John 2005; Cunha‐Cruz, Hujoel, and Kressin 2007; Ng and Leung 2006). Besides, it is confirmed that subgingival scaling and root planning significantly impact OHRQoL more than supragingival scaling (Goel and Baral 2017).

Various instruments have been validated to evaluate the OHRQoL, including the short and long versions of Oral Health Impact Profile‐14 and ‐49 (OHIP‐14 and OHIP‐49), Oral Impact on Daily Performance (OIDP), OHQoL‐UK(W), a conceptual instrument for pregnant women, etc. (McGrath and Bedi 2002; Slade 1997a, 1997b; Åstrøm and Okullo 2003; Fakheran et al. 2020). OHIP‐14 has been validated and is reliable for the Iranian population. It is a multidimensional measurement tool that examines the cultural, social, and functional aspects of quality of life (Navabi, Nakhaee, and Mirzadeh 2010).

Although fundamental concerns about the excessive use of antibiotics leading to the emergence of antibiotic resistance should be considered, we should not deprive patients of logical and tangible outcomes of antibiotic therapy (Loos and Needleman 2020). It should be noted that the absence of evidence does not mean that antibiotics cannot improve the patient's quality of life. Several international health policy and regulatory organizations have acknowledged the importance of PROs. If collected in a manner that adheres to scientific rigor, the results of studies focusing on patient‐centered outcomes have the potential to impact healthcare policy, pharmaceutical labeling claims, and clinical practice guidelines.

Based on the European Federation of Periodontology (EFP) S3‐level clinical practice guideline, the adjunctive use of specific systemic antibiotics can be considered in the treatment of generalized Stage III periodontitis in young adults. Accordingly, in this randomized controlled clinical trial, we aimed to evaluate the impact of systemic adjunctive antibiotic administration following subgingival instrumentation on the oral health‐related quality of life (OHRQoL) of young patients (≤ 40 years old) diagnosed with generalized Stage III periodontitis. To our knowledge, this is the first controlled clinical trial addressing this specific topic.

## 2 Materials and Methods

### 2.1 Clinical Trial Design

This prospective, randomized, controlled trial involved two parallel groups and included 70 patients undergoing nonsurgical periodontal treatment. This study received approval from the Ethics Committee of Isfahan University of Medical Sciences and was registered with the IRCT20201221049786N1 Registry of Clinical Trials on 13/02/2021. It adhered to the principles of the Declaration of Helsinki and followed CONSORT guidelines. Additionally, the Sex and Gender Equity in Research (SAGER) guidelines were adhered to in this investigation, and all participants provided written informed consent before their involvement.

Based on similar previous studies, to achieve 80% test power, 25 patients were required for each method to identify significant differences in median values at a 5% level (*d* = 0.7) (Navabi, Nakhaee, and Mirzadeh 2010; Pakpour et al. 2016; Hajian‐Tilaki et al. 2014). Since this study was organized during the COVID‐19 pandemic and there was a high possibility of patients not following the treatment protocol, we decided to include a significantly more significant number of participants. The ethics committee also approved this consideration. For this reason, we finally had 35 patients in each study group. The inclusion criteria were adult (≤ 18 and ≤ 40 years old) patients with generalized stage III periodontitis where clinical attachment loss was more than 3 mm in more than 30% of the remaining teeth and having at least 16 natural teeth (excluding third molars). Participants had no other oral diseases, except for periodontitis, such as decayed teeth, pericoronitis, soft tissue lesions, or malocclusion, that required treatment within the next 3 months. The exclusion criteria encompassed individuals with a prior allergic reaction to Penicillin or Metronidazole, those currently taking antibiotics, and individuals with systemic conditions like diabetes, HIV/AIDS, liver disorders, chronic renal failure, and autoimmune diseases. Also excluded were patients undergoing periodontal treatment in the preceding 6 weeks and individuals who were concurrently pregnant or breastfeeding. The reasons for excluding patients after inclusion in the study were the use of extra antibiotics, withdrawal of the process, and receiving any other dental treatment during the follow‐up period. These patients were selected by a periodontist and treated at the Periodontal Department of Isfahan University of Medical Sciences between February 2021 and April 2022.

After providing written informed consent, patients were randomly assigned to either the control (Placebo) or the test groups (Amoxicillin 500 mg and Metronidazole 250 mg, three times a day) for 7 days using computer‐generated randomization codes concealed in opaque, sealed envelopes. This study's randomization and subsequent allocation process involved a dental intern who prepared 70 medication packets. Each packet was assigned a unique numeric code ranging from 01 to 70, which was then printed on the labels of the respective packages. To ensure blinding, the medication packets were concealed with their numeric codes. When it came time to distribute the medication packets to eligible patients, they were given out in a numeric order. This means the patients received the medication based on the assigned numeric codes without knowing the contents. This process ensured that the distribution of the packets was done randomly and in a manner that maintained the blinding of both the dental intern and the patients. Hence, the investigator was blind to the treatment assigned to patients. The placebo was an inactive substance made by the Pharmacy School of Isfahan University of Medical Sciences and bore a resemblance to the primary drug.

Throughout the course of the medication period, an assistant called the patients three times a week to check on compliance with the consumption of antibiotics and placebos. After the medication week, the individuals were requested to return the bottles so that they could be examined for any leftover antibiotic or placebo tablets.

### 2.2 Evaluation of Efficacy

The clinical examination of each patient included Pocket Probing Depth (PPD), Bleeding On Probing (BOP), Clinical Attachment Loss (CAL), and Gingival Index (GI). For examiner calibration, a Kappa coefficient of 0.85 or higher was used. Ten patients, each having at least five teeth with PPD and CAL of 5 mm or more at proximal sites, were selected. Each patient underwent two examinations, with a 48‐h interval between the first and second assessments (Kappa = 0.89). PPD was measured using a HuFriedy PCP UNC 15 probe at four points around each tooth, including distobuccal/distolabial, mid buccal/midlabial, mesiobuccal/mesiolabial, palatal/lingual surfaces. The deepest PPD measurement was recorded for statistical analysis. CAL was calculated by adding the probing depth to the gingival margin at mid buccal/midlabial level, and the greatest CAL was the final record. The probe was gently inserted and moved around the teeth to test BOP. Then, 30 s after probing, the presence of BOP was determined, and the percentage of bleeding sites was scored. Based on the presence or absence of BOP for surfaces of all teeth, the GI was scored on a 0 –3 scale, with 0 indicating normal gingiva and 3 showing severe inflammation, redness, edema, spontaneous bleeding, and ulceration. These measurements were repeated at 1‐month and 3‐month recalls. A single dental intern blinded to the intervention recorded all the measures at all time points.

The same periodontist performed full‐mouth scaling and root planning using Cavitron Ultrasound Dental Scaling. Then, hand instrumentation was employed using a subgingival curette (Gracey curette SG 11/12, 5/6, or 13/14) to remove the deposits from the root and tooth surface for all patients at baseline. Additionally, all participants were given exact oral hygiene instructions (flossing and brushing in gentle circular strokes), fluoride‐rich toothpaste, dental floss, and medium‐bristle toothbrushes. Patients, operative investigator, and nonoperative investigator were all blinded to the medication received after the treatment procedure.

For the assessment of OHRQoL, each participant was asked to fill in the OHIP‐14 questionnaire at baseline and 1 month and 3 months later. The validity and reliability of the Persian version of OHIP‐14 had been previously confirmed among 400 participants (Cronbach's alpha = 0.85) (Navabi, Nakhaee, and Mirzadeh 2010). The patient's assessment was documented across seven categories: functional restrictions, physical unease, emotional distress, physical impairment, mental impairment, social limitations, and handicap in this survey. The scoring scale for this questionnaire spans from 0 to 56, with higher scores indicating a more unfavorable condition.

### 2.3 Statistical Analysis

Data analysis was performed using IBM SPSS statistical software, version 22.0, developed by IBM Corp. in Armonk, NY, USA. The initial data comparison utilized the Kruskal–Wallis test. Subsequently, the *t*‐test was employed to compare paired results across various groups. A significance level of *p* < 0.05 was deemed statistically meaningful.

## 3 Results

**Figure 1:**
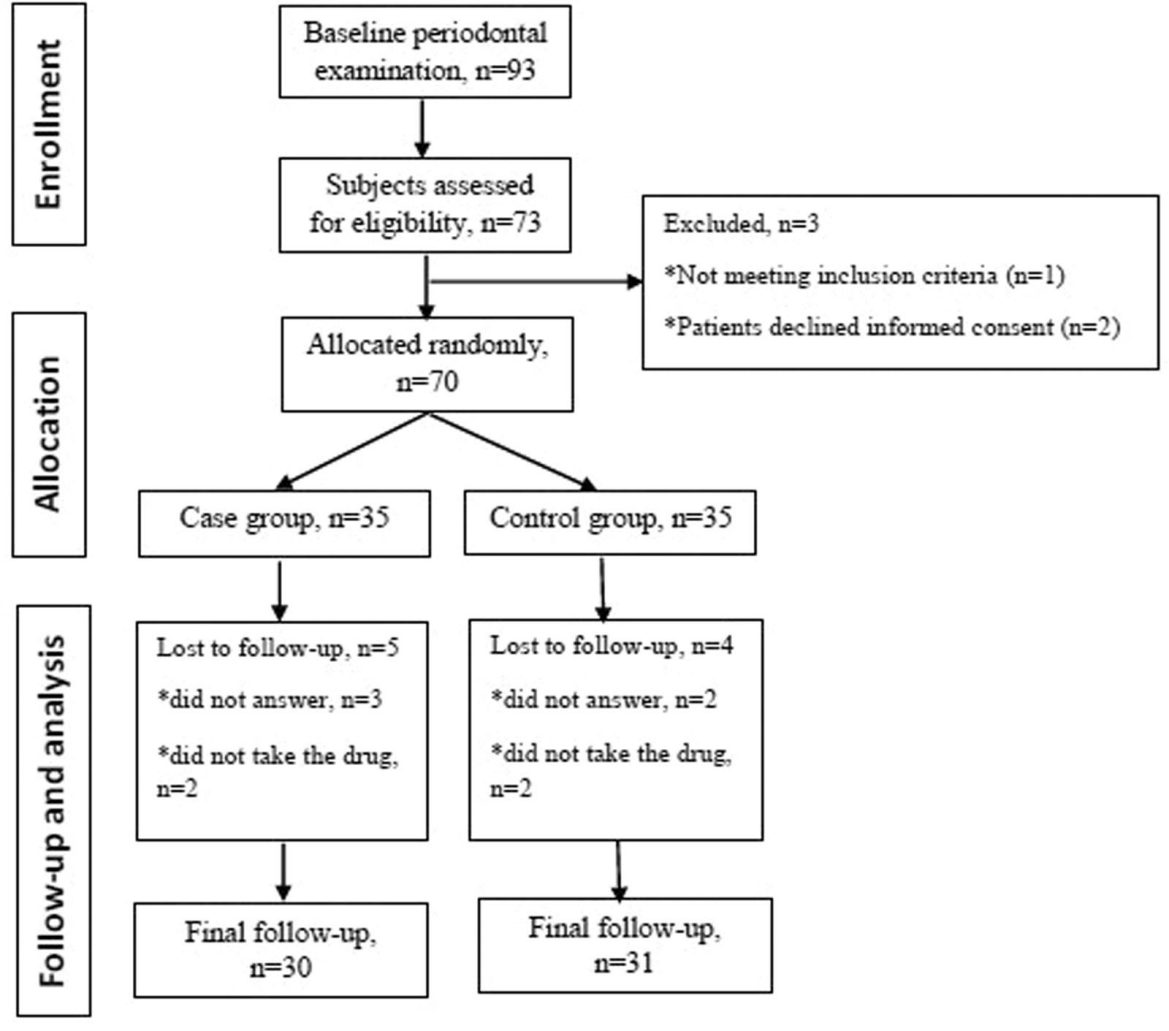
Flow chart of study.

In the test group, three patients did not answer the phone in the follow‐up period, and two patients informed us of bloating after taking the medication. In the control group, two patients did not answer the phone in the follow‐up period, and two forgot to take the medication, so they were excluded. Finally, the control group consisted of 16 (51.6%) men and 15 (48.4%) women with the mean age of 37.03 ± 11.02 years, while the test group included 16 (53.3%) men and 14 (46.7%) women with the mean age of 38.37 ± 10.63 years (Table 1, Figure 1). There was no significant difference between the two groups regarding age, gender, education level, and economic status

**Table 1:** Specification and comparison of patients' essential characteristics in two groups.

Characteristics		Control group (*n* = 31)	test group (*n* = 30)	*p* value
**Sex**	**Male**	16 (51.6%)	16 (53.3%)	0.893
	**Female**	15 (48.4%)	14 (46.7%)	
**Age, years**		37.03 ± 11.02	38.37 ± 10.63	0.632
**Education level**	**<=Diploma**	4 (13%)	9 (30%)	0.499
	**Bachelor's degree**	22 (71%)	16 (53.3%)	
	**Master's degree**	3 (9.6%)	4 (13.3%)	
	**PhD**	2 (6.4%)	1 (3.3%)	
**Economic status**	**Low income**	3 (9.7%)	4 (13.3%)	0.614
	**Middle income**	25 (80.6%)	21 (70%)	
	**Upper income**	3 (9.7%)	5 (16.7%)	

There was no significant difference between the two groups regarding the mean CAL, PPD, and GI at the beginning of the study and 1 and 3 months after the intervention (*p* value > 0.05). However, CAL and PPD in the test group and GI in both groups decreased significantly within 3 months (*p* value < 0.05). Moreover, the occurrence of bleeding on probing in the control group with 41.9% was far more than its occurrence in the test group with 10% 3 months after the intervention (*p *value = 0.008). In addition, although the mean of bleeding between the two groups was not significantly different in any of the studied times (*p* value > 0.05), a significant decrease in bleeding was observed in both groups 3 months after the intervention (*p* value < 0.05) (Table 2).

**Table 2:** Determination and comparison of the mean of CAL, PPD, GI, and BOP between the two groups.

Variables	Follow‐up	Control group (*n* = 31)	test group (*n* = 30)	*p* value*
CAL	Before intervention	5.09 ± 1.11	5.07 ± 1.17	0.918
	1 month after intervention	4.19 ± 0.94	3.97 ± 0.93	0.348
	3 months after intervention	3.84 ± 1.16	3.77 ± 0.82	0.780
*p* value**		0.393	0.031	
PPD	Before intervention	5.52 ± 1.18	5.57 ± 1.38	0.878
	1 month after intervention	3.62 ± 0.98	3.17 ± 1.05	0.093
	3 months after intervention	3.13 ± 0.92	3.10 ± 0.92	0.903
*p* value**		< 0.001	0.160	
BOP	Before intervention	31 (100%)	30 (100%)	—
	1 month after intervention	15 (48.4%)	11 (36.7%)	0.440**
	3 months after intervention	13 (41.9%)	3 (10%)	0.008**
*p* value**		< 0.001	< 0.001	
Volume of BOP, %	Before intervention	75.48 ± 15.88	73.67 ± 16.71	0.665
	1 month after intervention	19.03 ± 21.50	16.33 ± 22.36	0.633
	3 months after intervention	7.74 ± 10.55	5.67 ± 15.46	0.542
*p* value**		0.001	< 0.001	
GI	Before intervention	2.13 ± 0.34	2.13 ± 0.43	0.966
	1 month after intervention	1.32 ± 0.47	1.17 ± 1.38	0.163
	3 months after intervention	1.06 ± 0.25	1.07 ± 0.25	0.973
*p* value**		0.032	0.031	

Abbreviations: BOP, bleeding on probing; CAL, clinical attachment loss; GI, gingival index; PPD, pocket probing depth.

* The significance level obtained from the independent samples *t*‐test to compare the mean of the variables at any time between the two groups.

** The significance level obtained from the repeated measures ANOVA to compare the mean of the variables in each of the groups over time by adjusting patients' age, gender, education level, and economic status of the patients.

Furthermore, the evaluation of OHRQoL indicated no significant difference in the mean OHRQoL score between the two groups at the beginning of the study (*p* value > 0.05). However, the OHRQoL scores of the test group, with the mean scores of 8.7 ± 3.80 and 7.2 ± 3.21 1 month and 3 months after the intervention, respectively, were significantly lower than those of the control group, with the mean scores of 11.5 ± 4.59 and 9.5 ± 3.67, respectively (*p* value < 0.05). Although the mentioned decrease was not statistically significant (*p* value = 0.070), the OHRQoL score decreased significantly in the test group (*p* value < 0.001). The examination of each OHRQoL dimension revealed that physical pain, physical disability, and overall disability (Handicap) 1 and 3 months after the intervention and psychological discomfort only 3 months after the intervention were significantly lower in the test group as compared with the control group (*p* value < 0.001) (Table 3).

**Table 3:** Specification and comparison of the mean OHQoL and its dimensions between the two groups.

OHQoL	Follow‐up	Control group (*n* = 31)	test group (*n* = 30)	*p* value*
Total OHQoL	Before intervention	19.29 ± 8.63	20.93 ± 8.32	0.452
	1 month after intervention	11.52 ± 4.59	8.70 ± 3.80	0.012
	3 months after intervention	9.52 ± 3.67	7.27 ± 3.21	0.014
*p* value**		0.070	< 0.001	
Functional limitation	Before intervention	0.06 ± 0.25	0.07 ± 0.61	0.410
	1 month after intervention	0	0.03 ± 0.18	0.313
	3 months after intervention	0	0	—
*p* value**		0.963	0.368	
**Physical pain**	Before intervention	4.61 ± 1.99	4.73 ± 1.99	0.815
	1 month after intervention	2.68 ± 1.30	1.63 ± 0.24	0.002
	3 months after intervention	2.13 ± 1.26	1.23 ± 0.28	0.008
*p* value**		0.005	< 0.001	
**Psychological discomfort**	Before intervention	4.71 ± 1.46	4.73 ± 1.28	0.947
	1 month after intervention	4.13 ± 1.28	3.60 ± 1.13	0.094
	3 months after intervention	3.93 ± 1.26	3.30 ± 1.15	0.044
*p* value**		0.205	0.013	
**Physical disability**	Before intervention	4.16 ± 1.84	4.47 ± 1.79	0.515
	1 month after intervention	2.13 ± 1.59	0.77 ± 1.19	< 0.001
	3 months after intervention	1.22 ± 1.31	0.50 ± 1.04	0.020
*p* value**		0.026	< 0.001	
**Psychological disability**	Before intervention	2.68 ± 1.92	3.37 ± 1.92	0.166
	1 month after intervention	1.32 ± 1.01	1.53 ± 1.04	0.426
	3 months after intervention	1.00 ± 0.68	1.10 ± 1.03	0.655
*p* value**		0.447	0.002	
**Social disability**	Before intervention	0.97 ± 1.45	1.40 ± 1.30	0.226
	1 month after intervention	0.22 ± 0.50	0.47 ± 0.57	0.086
	3 months after intervention	0.29 ± 0.59	0.40 ± 0.56	0.460
*p* value**		0.988	0.051	
**Overall disability (Handicap)**	Before intervention	2.29 ± 0.86	2.03 ± 0.99	0.287
	1 month after intervention	1.13 ± 0.49	0.60 ± 0.49	< 0.001
	3 months after intervention	0.97 ± 0.55	0.57 ± 0.50	0.004
*p* value**		0.876	0.001	

Abbreviation: OHQoL, oral health‐related quality of life.

* The significance level obtained from the independent samples *t*‐test to compare the mean of the variables at any time between the two groups.

** The significance level obtained from the repeated measures ANOVA to compare the mean of the variables in each of the groups over time by adjusting patients' age, gender, education level, and economic status.

Finally, the evaluation of the relationship between OHRQoL and changes in CAL, PPD, and BOP indices in each of the studied groups showed that only PPD with correlation coefficients of 0.396 and 0.407 in the control and test groups, respectively, had a direct and significant relationship with OHRQoL (*p* value < 0.05) (Table 4).

**Table 4:** Evaluation of the relationship between OHQoL and CAL, PPD, and BOP in studied groups.

Group	Variables	Follow‐up	OHQoL	CAL	PPD	BOP	GI
**Control group**	**OHQoL**	Correlation coefficient	1				
		*p* value					
	CAL	Correlation coefficient	0.139	1			
		*p* value	0.456				
	PPD	Correlation coefficient	0.396	−0.165	1		
		*p* value	0.027	0.376			
	BOP	Correlation coefficient	−0.282	0.254	−0.036	1	
		*p* value	0.124	0.167	0.849		
	GI	Correlation coefficient	−0.093	0.066	−0.078	−0.039	1
		*p* value	0.620	0.725	0.676	0.836	
Test group	**OHQoL**	Correlation coefficient	1				
		*p* value					
	CAL	Correlation coefficient	0.308	1			
		*p* value	0.098				
	PPD	Correlation coefficient	0.407	0.133	1		
		*p* value	0.026	0.483			
	BOP	Correlation coefficient	−0.072	0.165	0.026	1	
		*p* value	0.707	0.382	0.891		
	GI	Correlation coefficient	0.122	−0.157	0.157	0.273	1
		*p* value	0.521	0.409	0.408	0.144	

Abbreviations: BOP, bleeding on probing; CAL, clinical attachment loss; GI, Gingival index; PD, pocket probing depth.

## 4 Discussion

The present study aimed to evaluate the effect of adjunctive antibiotics systemically administered to subgingival instrumentation on the OHRQoL of patients with periodontitis. Since there was no evidence in the literature, we conducted this trial on the participants with stage III periodontitis.

Based on the evidence for therapeutic management of periodontitis, mechanical debridement should be accompanied by antibiotic therapy to kill the remaining subgingival pathogens after conventional periodontal therapy (Winkelhoff, Rams, and Slots 1996). It is difficult to definitively decide about the usefulness of antibiotic therapy except for patients with deep pockets, progressive disease, or specific microbial patterns (Herrera et al. 2002). In contrast to previous studies, Hayes et al. showed that systemic tetracycline is not more beneficial than mechanical treatment alone (Hayes, Antczak‐Bouckoms, and Burdick 1992). Moreover, using antibiotics in patients with pockets of less than 4 mm is a highly controversial subject among studies (Elter et al. 1997). It is essential that all antibiotics can give rise to gastrointestinal effects (e.g., nausea, vomiting, diarrhea, abdominal pain, loss of appetite, bloating), and Amoxicillin, in particular, can cause secondary overgrowth of Candida species and *Clostridium difficile* infections (Mohsen, Dickinson, and Somayaji 2020). Due to the adverse effects and bacterial resistance, it is essential to consider the logical selection of antibiotics to optimize medication efficacy (Keestra et al. 2015; Kapoor et al. 2012). The adjunctive use of 250 mg Metronidazole plus 500 mg Amoxicillin (three times a day) 7 days after nonsurgical periodontal therapy showed statistically significant and clinically relevant benefits for periodontitis patients (McGowan, McGowan, and Ivanovski 2018; Borges et al. 2017). The evaluation of treatment outcomes has traditionally been dominated by objective clinical outcome measures that, although necessary, cannot demonstrate the patients' perception and priorities regarding the treatment (Loos and Needleman 2020). Hence, prominent international health policy, regulatory bodies, and patients acknowledge the significance of patient‐reported outcomes (Patrick et al. 2007; Doward, Gnanasakthy, and Baker 2010).

During the last decade, researchers have focused on measuring the quality of life besides clinical indices such as PPD, CAL, and BOP due to the importance of this issue. Although there is no final definition for OHRQoL, it is described as the subjective evaluation of a person's satisfaction with care and self‐concept (Sischo and Broder 2011). It is improbable for medical technology to capture all the information regarding treatment or the illness; this type of data can be acquired solely from the patient. Consequently, a growing focus is on assessing patient‐reported outcomes (Chin and Lee 2008). Furthermore, in some diseases, patient‐reported outcome measurements are more valuable and play a key role, especially when the primary objective of the treatment is not solely centered on survival (Singh 2010; Deshpande et al. 2011). As a result of greater attention to OHRQoL, various measurement tools have been developed, such as OHIP‐14, a shorter version of the OHIP‐49 questionnaire (Slade 1997a; Organization 1980). The OIDP scale assesses the effect on individuals' daily lives and is convenient for use in population surveys (Åstrøm and Okullo 2003; Adulyanon, Vourapukjaru, and Sheiham 1996). Robinson et al. compared the concurrent validity of OHIP‐14 and OIDP. Based on the results, although completion rates were similar, OIDP indicated a total score due to the severe skewness of the data (Robinson et al. 2001). Hence, OHIP‐14 was considered the instrument of choice to assess OHRQoL in this study.

Locker et al. reported a five‐score reduction in the mean OHIP‐14 score, representing a significant difference (Locker and Allen 2007). In the present study, the mean OHIP‐14 decreased from 20.93 ± 8.32 at baseline to 8.70 ± 3.80 after 1 month and 7.27 ± 3.21 after 3 months of follow‐up in the test group, which was statistically significant. In the control group, it decreased from 19.29 ± 8.63 at baseline to 11.52 ± 4.59 and 9.52 ± 3.67 1 month and 3 months after intervention, respectively, which was not statistically significant compared to the test group. Bery et al. investigated the association between oral health status and HQoL. They reported a mean OHIP‐14 score of 8.6 for subjects over 15 years (mean age of 34.7), which is undeniably lower than the mean score obtained in the present study at baseline. The variation could be attributed to diverse economic and cultural backgrounds (Bery et al. 2009). The results of this study were in line with other studies in that physical pain and psychological discomfort indicated the highest scores using OHIP‐14 (Meusel et al. 2015; Mendez et al. 2017; Sonnenschein et al. 2018). Wong et al. reported a significant reduction in mean OHIP‐14 score (from 17 at baseline to 14 after 6 months of follow‐up) after nonsurgical periodontal treatment, which is in line with the results of another study that showed nonsurgical periodontal treatment might improve the patients' OHRQoL (Åslund et al. 2008; Wong et al. 2012).

Following the most recent clinical practice guideline issued by the EFP, they do not recommend the routine use of antibiotics administered systemically alongside subgingival instrumentation for patients with periodontitis (Sanz et al. 2020). The basis for this recommendation stems from a high‐quality systematic review published in 2020 (Teughels et al. 2020). In this review, the authors analyzed 34 articles (of which 28 were clinical trials) to assess the clinical effectiveness of systemic antimicrobial agents used adjunctively in periodontitis patients. Interestingly, none of the studies included in this review addressed aspects like patients' OHRQoL or their satisfaction with the treatment process and outcomes (Teughels et al. 2020). Consequently, due to the lack of such data, the EFP couldn't incorporate patient‐reported results into their clinical practice recommendations for antibiotic use in managing periodontitis Stages I–III (Sanz et al. 2020). Furthermore, another consensus report by the authors revealed a similar gap, indicating no available data on how the systemic administration of antibiotics during nonsurgical periodontal therapy impacts OHRQoL (Pretzl et al. 2019).

To the best of our knowledge, the impact of adjunctive antibiotics on OHRQoL after nonsurgical periodontal therapy in conjunction with clinical outcomes has yet to be studied, and this study was the first randomized controlled trial to investigate this issue based on the EFP S3‐level clinical practice guideline. Various studies have focused on factors that affect the OHRQoL of patients with periodontitis, including untreated dental decay, destroyed root surfaces, malocclusion, social and emotional states, etc (Sischo and Broder 2011; Broder, Wilson‐Genderson, and Sischo 2012; Andersson et al. 2010). Additionally, Peikert et al. suggested that adjunctive use of antibiotics could positively affect OHRQoL. Nevertheless, this study was not controlled, and given the limitations, the results should be interpreted with caution (Peikert et al. 2019). Besides, Harks et al. evaluated the effect of antibiotics on OHRQoL as a secondary outcome, but there was no correlation between the studied clinical parameters and OHQoL (Harks et al. 2015).

In line with the present results, other studies have shown a direct association between PPD and OHRQoL, i.e., patients with a lower PPD underwent significant improvement in OHRQoL (Needleman et al. 2004; Brauchle, Noack, and Reich 2013). Theodoridis et al. evaluated the impact of surgical and nonsurgical periodontal treatments on OHQoL in conjunction with clinical parameters in Greek adults. Although there was a significant improvement in clinical parameters and OHRQoL after nonsurgical periodontal therapy, surgical treatments did not improve OHRQoL. Moreover, in contrast to the results of the present study, no correlation was found between OHRQoL and clinical parameters. This study was uncontrolled, and the different methodologies adopted can explain this contrast (Theodoridis et al. 2020). In addition, some other studies have confirmed that the correlation between clinical parameters and OHRQoL is questionable (Peikert et al. 2019). Different intervals between follow‐ups and disparate age groups and using different indices offer a rational explanation for these results (Andersson et al. 2010; Gil‐Montoya et al. 2008; Saito et al. 2010).

The assessment of antibiotics' effect on clinical indicators revealed that, in the test group, the average BOP decreased significantly after 3 months compared to the control group, aligning with results from prior studies (Keestra et al. 2015; Haffajee, Socransky, and Gunsolley 2003). In line with previous research, the test group's average CAL and PPD scores showed significant improvement compared to the control group. However, it is worth noting that our study had a smaller participant pool, which may explain the contrasting outcomes observed in this research (Keestra et al. 2015; Harks et al. 2015; Haffajee, Socransky, and Gunsolley 2003).

In conclusion, while this study highlights the potential benefits of systemic administration of Amoxicillin 500 mg and Metronidazole 250 mg, three times a day, as adjunctive therapy to periodontal mechanical treatment, the findings must be interpreted within the limitations of the study design.

The primary limitation of this study is the relatively short follow‐up period, which was necessitated by the unplanned suspension of activities during the COVID‐19 pandemic. A longer‐term follow‐up of these patients would be crucial to assess whether the additional benefits observed with antibiotic therapy, when used as an adjunct to subgingival instrumentation, are maintained over time. Extending the duration of the study would provide a more comprehensive understanding of the sustainability of these therapeutic effects and could offer valuable insights into the long‐term efficacy of this combined treatment approach.

Furthermore, the OHIP‐14 questionnaire, while widely used to assess oral health‐related quality of life, has inherent limitations (Campos et al. 2021). Being a self‐reported measure, it is subject to individual biases, including patients' subjective interpretation of their symptoms and personal expectations of treatment outcomes. These biases can influence the accuracy and reliability of the data, especially in studies evaluating clinical interventions. Additionally, the OHIP‐14 may not fully capture the nuances of specific clinical conditions or the long‐term effects of therapeutic interventions, which could lead to an underestimation or overestimation of treatment impacts.

Therefore, future studies should take these limitations into account by incorporating more objective clinical outcomes alongside patient‐reported measures. It is important for researchers and clinicians to be cautious when interpreting self‐reported outcomes and to consider supplementing the OHIP‐14 with additional tools or extended follow‐up periods to achieve a more comprehensive understanding of the long‐term effects of treatment.

## Author Contributions

**Parastoo Parhizkar:** conceptualization, methodology, formal analysis, investigation, software, writing–original draft. **Jaber Yaghini:** conceptualization, methodology, formal analysis, investigation, writing–review and editing, supervision. **Omid Fakheran:** conceptualization, formal analysis, investigation, writing–review and editing, supervision. All authors read and approved the final version of the article.

## Ethics Statement

This study received approval from the Ethics Committee of Isfahan University of Medical Sciences (IR. MUIRESEARCH. REC.1399.572).

## Consent

Written informed consent was obtained from all participants.

## Conflicts of Interest

The authors declare no conflicts of interest.

## Data Availability

The data that support the findings of this study are available from the corresponding author upon reasonable request.
